# Molecular Underpinnings of Nitrite Effect on CymA-Dependent Respiration in *Shewanella oneidensis*

**DOI:** 10.3389/fmicb.2016.01154

**Published:** 2016-07-21

**Authors:** Miao Jin, Huihui Fu, Jianhua Yin, Jie Yuan, Haichun Gao

**Affiliations:** Institute of Microbiology and College of Life Sciences, Zhejiang UniversityHangzhou, China

**Keywords:** *Shewanella*, nitrite, regulation, inhibition, respiration

## Abstract

*Shewanella* exhibit a remarkable versatility of respiration, with a diverse array of electron acceptors (EAs). In environments where these bacteria thrive, multiple EAs are usually present. However, we know little about strategies by which these EAs and their interaction affect ecophysiology of *Shewanella*. In this study, we demonstrate in the model strain, *Shewanella oneidensis* MR-1, that nitrite, not through nitric oxide to which it may convert, inhibits respiration of fumarate, and probably many other EAs whose reduction depends on quinol dehydrogenase CymA. This is achieved via the repression of cyclic adenosine monophosphate (cAMP) production, a second messenger required for activation of cAMP-receptor protein (Crp) which plays a primary role in regulation of respiration. If nitrite is not promptly removed, intracellular cAMP levels drop, and this impairs Crp activity. As a result, the production of nitrite reductase NrfA, CymA, and fumarate reductase FccA is substantially reduced. In contrast, nitrite can be simultaneously respired with trimethylamine *N*-oxide, resulting in enhanced biomass.

## Introduction

*Shewanella oneidensis* MR-1 is a Gram-negative facultative anaerobe with remarkable respiration abilities that permit the use of a diverse array of terminal electron acceptors (EAs), including fumarate, nitrate, nitrite, thiosulfate, trimethylamine *N*-oxide (TMAO), dimethylsulfoxide (DMSO), Fe(III), Mn(III) and (IV), Cr(VI), and U(VI), among others (Fredrickson et al., 2008). As anticipated there are a large number of respiratory pathways, some of which have been elucidated over the past two decades (Gon et al., 2002; Maier et al., 2003; Gralnick et al., 2006; Cruz-Garcia et al., 2007; Gao et al., 2009; Shirodkar et al., 2011; Shi et al., 2012). For nitrate and nitrite respiration, most bacteria use the quinol dehydrogenases NapC and NrfBCD (or NrfH) to transfer electrons to the terminal reductases NapA and NrfA, respectively. In contrast, *S. oneidensis* lacks these dehydrogenases (Jepson et al., 2006; Gao et al., 2009; Simpson et al., 2010; Fu et al., 2014). Instead, CymA, a cytoplasmic-membrane-bound cytochrome *c*, is recruited to function for the missing proteins (Marritt et al., 2012). The role of CymA could be partially, but significantly less effectively, fulfilled by the *bc*_1_ complex and SirCD, which primarily function as quinol dehydrogenases to transport electrons to the cytochrome *cbb*_3_ oxidase and to sulfite reductase, respectively (Cordova et al., 2011; Zhou et al., 2013; Fu et al., 2014). Interestingly, only two components are needed for the formation of functional NAP and NRF complexes, CymA-NapA and CymA-NrfA (Gao et al., 2009). One consequence of sharing CymA is that reduction of nitrite to ammonium by NrfA does not commence until nitrate is thoroughly exhausted, resulting in a characteristic two-step reduction of nitrate (Gao et al., 2009).

In addition to being EAs for respiration, nitrate (as a precursor to nitrite) and nitrite have been used for centuries as preservatives in meat products to inhibit growth of bacterial pathogens. The antimicrobial action of nitrite is generally attributed to the formation of nitric oxide (NO), which interferes with protein cofactors, such as Fe–S clusters, heme, and lipoamide, or promotes the formation of reactive nitrogen species (Reddy et al., 1983; Hyduke et al., 2007; Mason et al., 2009; Richardson et al., 2011). In *S. oneidensis*, the cytochrome *cbb*_3_ oxidase, the enzyme complex predominantly responsible for oxygen respiration, is the primary target of nitrite stress (Fu et al., 2013; Zhou et al., 2013; Yin et al., 2015). This corresponds to the fact that the *Escherichia coli* cytochrome *bo* terminal oxidase is highly susceptible to NO (Mason et al., 2009).

When nitrite is added to *S. oneidensis* cultures grown under aerobic conditions, cells are constantly under nitrite threat until entry into the stationary phase, when reduction of nitrite to non-harmful ammonium ion occurs (Dong et al., 2012; Zhang et al., 2013). In contrast, when oxygen is absent, nitrite can be immediately consumed as an EA, leading to a rapid decrease in its concentrations (Gao et al., 2009). Despite this, levels at which nitrite completely prevents growth are ~25 and ~5 mM for aerobic and anaerobic growth (nitrite as sole EA) respectively (Dong et al., 2012; Zhang et al., 2013), implying that nitrite is more toxic anaerobically than aerobically. One handy explanation is that nitrite is converted to NO faster under anoxic conditions. In parallel, nitrite may be attacking a cell process that is not, or less, necessary for aerobic growth.

In this study, we examined the role of nitrite during anaerobiosis and found that inhibition of growth on fumarate, probably many other CymA-dependent EAs, by nitrite is associated with *c*yclic *a*denosine 3′,5′-*m*ono*p*hosphate (cAMP). In bacteria, the physiological function of cAMP signaling, which has been extensively studied for decades, is diverse (Green et al., 2014). For regulation of metabolism, cAMP as an effector forms a regulatory system with *c*AMP *r*eceptor *p*rotein (Crp) to coordinate the allocation of proteomic resources with different metabolic demands in different nutrient environments (You et al., 2013). In *S. oneidensis*, the cAMP-Crp system is the primary regulator mediating respiration of various EAs, as well as many other biological processes (Saffarini et al., 2003; Dong et al., 2012; Fu et al., 2013; Zhou et al., 2013; Gao et al., 2015). Proper cAMP levels must be maintained, by adenylate cyclases (ACs) and phosphodiesterase for synthesis and degradation respectively (Charania et al., 2009; Yin et al., 2016), because either too much or too little negatively impacts aerobic growth (Zhou et al., 2013; Yin et al., 2016). We further showed that nitrite via a yet unknown mechanism triggers repression of cAMP production, by which nitrite eventually compromises Crp activation, leading to substantial decreases in the production of NrfA, CymA, and the fumarate reductase, FccA.

## Materials and Methods

### Bacterial Strains, Plasmids, and Culture Conditions

The bacterial strains and plasmids used in this study are listed in **Table 1**. In-frame deletion strains derived from *S. oneidensis* MR-1 used in this study were constructed and verified in previous reports. Sequences of the primers used in this study are available upon request. *E. coli* and *S. oneidensis* were grown aerobically in Lysogeny broth (LB, Difco, Detroit, MI, USA) at 37 and 30°C for genetic manipulation. When appropriate, the growth medium was supplemented with the following: 2, 6-diaminopimelic acid (DAP), 0.3 mM; ampicillin, 50 μg/ml; kanamycin, 50 μg/ml; gentamycin, 15 μg/ml; and streptomycin, 100 μg/ml. All chemicals were obtained from Sigma-Aldrich (St. Louis, MO, USA) unless otherwise noted.

**Table 1:** Strains and plasmids used in this study.

Strain or plasmid	Description	Reference or source
Strain		
*Escherichia coli*		
DH5α	Host for cloning	Lab stock
WM3064	Donor strain for conjugation, Δ*dapA*	W. Metcalf, UIUC
*Shewanella oneidensis*		
MR-1	Wild type	Lab stock
HG0970	Δ*fccA* derived from MR-1	Gao et al., 2010a
HG3286-4	Δ*cyd* derived from MR-1	Chen et al., 2015
HG3980	Δ*nrfA* derived from MR-1	Gao et al., 2009
HG3982	Δ*narP* derived from MR-1	Dong et al., 2012
HG4951	Δ*cymA* derived from MR-1	Gao et al., 2009
Plasmid		
pHGC01	Integrative vector for complementation	Fu et al., 2013
pHGEI01	Integrative *E. coli lacZ* reporter vector	Fu et al., 2014
pBBR-Cre	Helper vector for antibiotic marker removal	Fu et al., 2013
pHGE-P*tac*	Km^r^, IPTG-inducible P*_tac_* expression vector	Luo et al., 2013
pHGE-P*tac*-*cymA*	Inducible expression of *cymA*	This study
pHGE-P*tac*-*fccA*	Inducible expression of *fccA*	This study
pHGE-P*tac*-*nrfA*	Inducible expression of *nrfA*	This study
pHGE-P*tac*-*gfp*	Inducible expression of *gfp*	This study
pHGE-P*tac-scyA*	Inducible expression of *scyA*	This study
pHGEI-P*crp-lacZ*	*E. coli lacZ* under control of *crp* promoter	This study
pHGEI-P*cyaA-lacZ*	*E. coli lacZ* under control of *cyaA* promoter	This study
pHGEI-P*cyaC-lacZ*	*E. coli lacZ* under control of *cyaC* promoter	This study
pHGEI-P*cpdA-lacZ*	*E. coli lacZ* under control of *cpdA* promoter	This study

Growth of *S. oneidensis* strains under aerobic or anaerobic conditions was measured at 600 nm (OD_600_). MS defined medium containing 30 mM lactate as electron donor was used as previously described (Shi et al., 2015). For aerobic growth, mid-log phase cultures (~0.2 of OD_600_) were inoculated into fresh medium to ~0.02 and shaken at 200 rpm at 30°C. For anaerobic growth, mid-log phase aerobic cultures were pelletted by centrifugation, purged with nitrogen, suspended in fresh medium prepared anaerobically to an OD_600_ of ~0.02. EAs used in this study include nitrite (2 mM), fumarate (20 mM), and TMAO (20 mM). To assay the effect of NO on growth, DETA NONOate (t_1/2_, 20 h at 37°C and 56 h at 25°C) was used because it releases NO slowly and can maintain a relatively steady NO concentration (Zhang et al., 2013). For NO scavenging, carboxy-PTIO (Invitrogen, Carlson, CA, USA) was added to a final concentration of 0.1 mM. For chemical complementation, cAMP of various levels was used.

### Controlled Expression of *cymA*, *fccA*, *nrfA*, and *scyA* Genes

To assess effects of the four genes expressed at varying levels on nitrite-associated physiology, we placed each of them under the control of isopropyl-β-D-thiogalactopyranoside (IPTG)-inducible promoter P*_tac_* within pHGE-P*tac* (Luo et al., 2013). After verification by sequencing, the vectors were transferred into the relevant strains via conjugation. Cells carrying vectors of interest were grown in media indicated in the text and/or figure legends in the presence of IPTG of varying levels.

### Chemical Assays

Concentrations of nitrite in culture supernatants were measured by a modified Griess assay and quantitated spectrophotometrically at 540 nm (Miranda et al., 2001). Intracellular cAMP concentrations were measured using a commercially available kit (cAMP direct immunoassay kit; BioVision) essentially the same as described before (Yin et al., 2016). Standard curves were made with commercial agents each time.

### Promoter Activity Assay

The activity of various promoters was assessed using a single-copy integrative *lacZ* reporter system as described previously (Fu et al., 2014). A fragment containing the sequence upstream of each operon from -300 to +1 (relative to the translation start codon) was amplified and cloned into the reporter vector pHGEI01 and verified by sequencing, These plasmids were then transferred by conjugation into relevant *S. oneidensis* strains. Plasmid pHGEI01 containing promoters of interest integrates into the chromosome and the antibiotic marker is then removed by an established approach (Fu et al., 2013, 2014). Cells grown to the mid-log phase were collected and β-galactosidase activity assays were performed with an assay kit as described previously (Jin et al., 2013).

### Expression of GFP Fusions and Quantitation of Fluorescence

To validate protein overproduction driven by the IPTG-inducible P*tac*, constructs expressing GFP protein were prepared. After verification by sequencing, the vectors were moved into relevant *S. oneidensis* strains by conjugation. Expression of GFP fusions was visualized using a confocal microscope as described previously (Luo et al., 2013). For quantitation, mid-log phase cultures were collected, washed with phosphate-buffered saline containing 0.05% Tween 20, and resuspended in the wash buffer to an OD_600_ of 0.1. 100 μl of the cell suspensions were transferred into black 96-well plates at various time intervals and fluorescence was measured using a fluorescence microplate reader (M200 Pro Tecan) with excitation at 485 nm and detection of emission at 515 nm.

### SDS-PAGE, Heme-Staining, and Immunoblotting Assays

Unless otherwise noted, mid-log phase cells were harvested, washed with phosphate buffered saline (PBS), resuspended in the same buffer, and sonicated. Protein concentrations of the cell lysates were determined by the bicinchoninic acid assay (Pierce Chemical). The cell lysates were resolved by SDS-PAGE using 12% polyacrylamide gels and stained with 3,3′,5,5′-tetramethylbenzidine (TMBZ) as described elsewhere (Thomas et al., 1976).

Immunoblotting analysis was performed essentially as previously described (Dong et al., 2012). Proteins separated by SDS-PAGE were electrophoretically transferred to polyvinylidene difluoride (PVDF) membranes according to the manufacturer’s instructions (Bio-Rad). The gels were blotted for 2 h at 60 V using a Criterion blotter (Bio-Rad). The blotting membrane was probed with a rabbit polyclonal antibody against *S. oneidensis* Crp (Dong et al., 2012). Goat anti-rabbit IgG-HRP (horseradish peroxidase; Roche Diagnostics) was used as the secondary antibody (1:5,000) and the signal was detected using a chemiluminescence Western blotting kit (Roche Diagnostics) in accordance with the manufacturer’s instructions. Images were visualized with a UVP imaging system.

### Nitrite Sensitivity Assay

*Shewanella oneidensis* strains grown to an OD_600_ of ~0.4 were adjusted to approximately 10^7^ CFUs/ml, followed by 10-fold serial dilutions. Ten microliters of each dilution was spotted onto LB plates containing 3 mM nitrite. The plates were incubated at 30°C before being read. The assays were repeated at least three times with similar results.

### Other Analyses

LC/MS/MS analyses of excised proteins were carried out essentially as described previously (Sun et al., 2013). Student’s *t-*test was performed for pairwise comparisons. Values are presented as mean ± SD in the relevant figures.

## Results

### Inhibitory Effect of Nitrite on Anaerobic Growth of *S. oneidensis*

**FIGURE 1:**
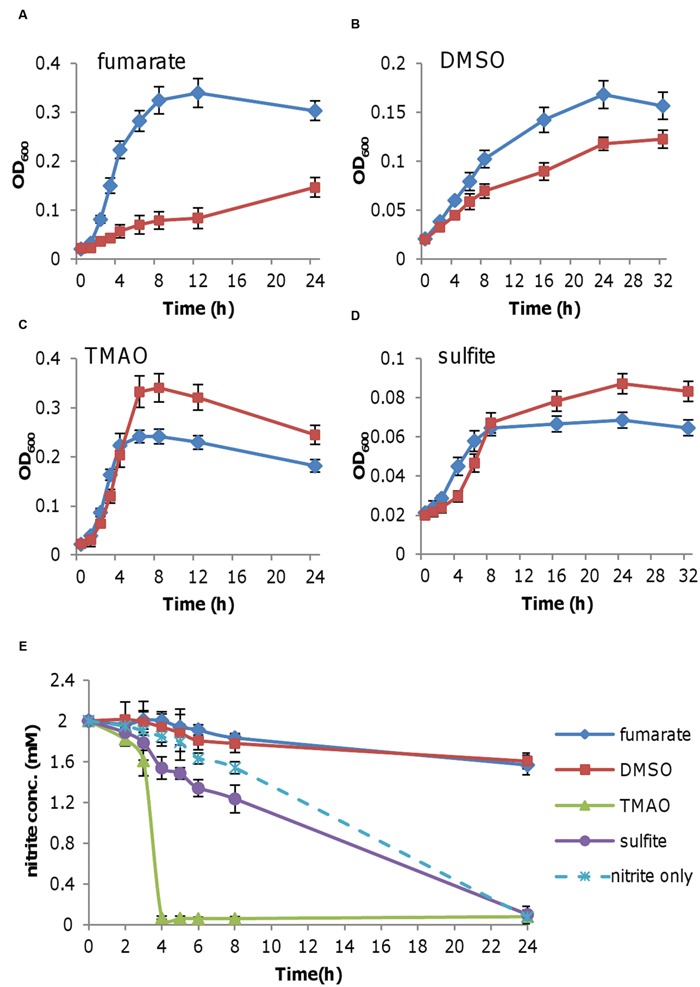
**Effect of nitrite on the growth of *Shewanella oneidensis* with various EAs.** Mid-log phase cultures (~0.2 of OD_600_) grown under aerobic conditions were pelletted, purged with nitrogen, washed twice with EA-free medium prepared under anaerobic conditions, and then inoculated into fresh media containing the indicated EAs, without (blue diamond) or with 2 mM nitrite (red square). **(A)** Medium containing 20 mM fumarate. **(B)** 20 mM DMSO. **(C)** 20 mM TMAO. **(D)** 20 mM sulfite. **(E)** Nitrite concentrations in cultures from **(A–D)**. Nitrite as the sole EA (nitrite only) was included for comparison. At the indicated time points, samples were taken for the determination of nitrite concentrations. All experiments were performed at least three times with standard deviations presented as error bars.

*Shewanella oneidensis* grows extremely poorly on nitrite as sole EA (Zhang et al., 2013). Two mM nitrite facilitates growth most effectively, resulting in an OD_600_ up to 0.06, which is barely visible (Cruz-Garcia et al., 2007; Gao et al., 2009). To determine whether this poor growth is a result of nitrite toxicity, we measured growth of *S. oneidensis* on fumarate, DMSO, TMAO, or sulfite in the presence of 2 mM nitrite. While the former two rely on CymA for electrons, the remaining are CymA-independent (Schwalb et al., 2003). As shown in **Figure 1A**, growth with fumarate was substantially retarded in the presence of nitrite, manifesting that nitrite inhibits fumarate respiration. Inhibition by nitrite was also observed with DMSO, albeit less significantly (**Figure 1B**). In contrast to fumarate and DMSO, nitrite had a negative impact on the early phase of growth with TMAO or sulfite but higher cell densities were eventually obtained (**Figures 1C,D**). We reasoned that this difference may be due to the reduction of nitrite. Thus we measured the remaining nitrite in supernatants of cultures grown on nitrite with one of these EAs. Consistent with the growth phenotype, nitrite reduction in cells grown on fumarate or DMSO hardly occurred, whereas nitrite was consumed in the presence of TMAO or sulfite (**Figure 1E**). In fact, TMAO dramatically promoted reduction of nitrite as the latter was depleted in 4 h. It was worth noting that compared to fumarate and TMAO, DMSO and sulfite were poor EAs to support growth, making it difficult to assess influence of nitrite; hence, we used fumarate and TMAO as EAs for subsequent experiments through this study. As impacts of nitrite on respiration of different EAs vary, the data suggest that nitrite toxicity may not be the only reason explaining inhibition of growth on fumarate or DMSO.

### Nitrite Inhibition of Fumarate Respiration is Not via NO

**FIGURE 2:**
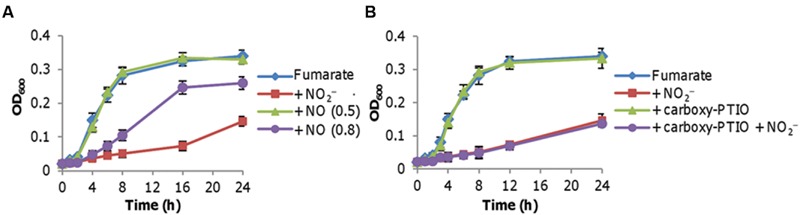
**Nitrite inhibition of fumarate respiration is not via NO.** Mid-log phase cultures (~0.2 of OD_600_) were inoculated into the indicated media. Fumarate, 20 mM; nitrite, 2 mM. **(A)** Growth of the wild-type on fumarate in the absence and presence of NO. NO was released from DETA NONOate at concentrations ranging from 0.1 to 0.8 mM. Results from 0.5 to 0.8 mM are shown as representatives of ‘no-effect’ (≤0.5 mM) and ‘significant inhibition’ (>0.6 mM), respectively. **(B)** Effect of carboxy-PTIO on growth of the wild-type. Carboxy-PTIO was added to a final concentration of 0.1 mM, which is sufficiently high to scavenge endogenous NO. Experiments were performed at least three times with error bars representing the standard deviation.

Previously we had shown that an *S. oneidensis* strain lacking the cytochrome *bd* oxidase is highly susceptible to nitrite but not to NO under aerobic conditions, implicating a difference in toxicities of nitrite and NO to cells grown aerobically (Fu et al., 2013; Zhang et al., 2013). Despite this, whether the inhibitory effect of nitrite is due to the production of NO requires further validation given that *S. oneidensis* produces NO endogenously in the presence of either nitrate or nitrite under anaerobic conditions only (Price et al., 2007). To this end, we first examined the growth of *S. oneidensis* on fumarate in the presence of 0.1–0.8 mM DETA NONOate (NO generating agent; **Figure 2A**). Up to 0.5 mM DETA NONOate did not inhibit growth, but at higher concentrations caused a significantly reduced growth rate and final biomass yield. Given that the concentration of NO generated endogenously is no more than 50 nM (Plate and Marletta, 2012), it is unlikely that it is the inhibitor. Nevertheless, as a further confirmation we examined the effect of carboxy-PTIO, a NO radical scavenger (Goldstein et al., 2003). Carboxy-PTIO at 0.1 mM, which had no noticeable effects on growth with either oxygen or fumarate, did not reverse the nitrite inhibition of fumarate-supported growth (**Figure 2B**). Based on these data we conclude that nitrite rather than NO accounts for the inhibition of growth on fumarate.

### Neither CymA nor the Fumarate Reductase Is Inhibited by Nitrite

**FIGURE 3:**
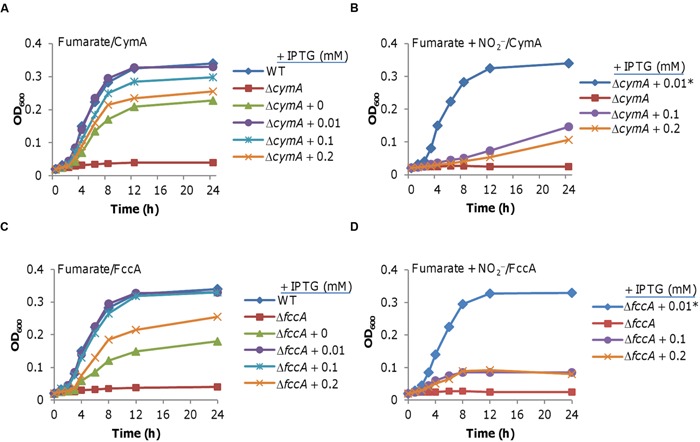
**Neither CymA nor FccA is inhibited by nitrite.** Expression of the *cymA* and *fccA* genes was driven by P*_tac_*, which is under the control of IPTG, within pHGE-P*tac*. In this figure and all others, the numbers shown represent the amounts of IPTG used for induction. Strains without a number contain just the vector, and serve as the control. **(A)** Growth of an *S. oneidensis* Δ*cymA* mutant producing CymA at varying levels in the absence of nitrite. **(B)** Growth as in A but in the presence of nitrite. Growth of the Δ*cymA* mutant with 0.01 mM IPTG without nitrite (Δ*cymA* + 0.01*) was included for comparison. **(C)** Growth of an *S. oneidensis* Δ*fccA* strain producing FccA at varying levels in the absence of nitrite. **(D)** Growth as in C but in the presence of nitrite. Growth of the Δ*fccA* strain with 0.01 mM IPTG but without nitrite (Δ*fccA* + 0.01*) was included for comparison. In all panels, error bars representing standard deviations from at least three independent experiments, similar to those shown in **Figures 1** and **2**, were omitted for clarity.

As mentioned earlier, in addition to terminal reductases the pathways for fumarate and TMAO reduction differ in that they employ distinct quinol dehydrogenases, CymA and TorC, respectively (Schwalb et al., 2003). To evaluate whether CymA may be a target of nitrite inhibition, we placed the *cymA* gene under the control of the IPTG-inducible promoter P*_tac_* and determined whether its overexpression suppresses the nitrite inhibition of fumarate-supported growth (Luo et al., 2013; Shi et al., 2014; **Figure 3A**). As expected, the Δ*cymA* strain hardly grew on fumarate (Schwalb et al., 2003). While complete complementation was obtained with 0.01 mM IPTG, but without IPTG addition (low expression as P*_tac_* is slightly leaky (Luo et al., 2013; Shi et al., 2014; Sun et al., 2014; Fu et al., 2015) or with higher concentrations there was only partial rescue of the growth defect, indicating that CymA in excess has a negative effect on growth (**Figure 3A**). Importantly, overproduction of CymA did not alleviate nitrite inhibition. Rather it showed a similar negative impact on growth of the Δ*cymA* strain in the presence of nitrite. These data rule out the possibility that CymA is one of the primary targets of nitrite (**Figure 3B**).

We then tested whether or not fumarate reductase FccA is inhibited by nitrite using the same protocol. Similar results were obtained, including a dose-dependent complementation and a negative effect of excess FccA with or without nitrite (**Figures 3C,D**). To test the possibility that the growth defect resulting from CymA or FccA overproduction is a protein-burden effect, we repeated the experiment with the *gfp* gene under the same conditions. Overproduction of GFP did not impair growth (Supplementary Figure S1). Furthermore, Given that both CymA and FccA are *c*-type cytochromes we overproduced periplasmic *c*-type cytochrome ScyA to determine whether the phenotype is specific to any *c*-type cytochrome when in excess. Excess ScyA induced by 0.2 mM IPTG increased nitrite resistance (Supplementary Figure S1), which is in agreement with a previous study (Yin et al., 2015). However, it did not inhibit growth on fumarate in the absence of nitrite (Supplementary Figure S1), indicating that growth inhibition by excess CymA or FccA may not be applicable to other *c*-type cytochromes. Overall, these data indicate that neither CymA nor FccA is directly inhibited by nitrite.

### Nitrite Alters the Content of *c*-Type Cytochromes

**FIGURE 4:**
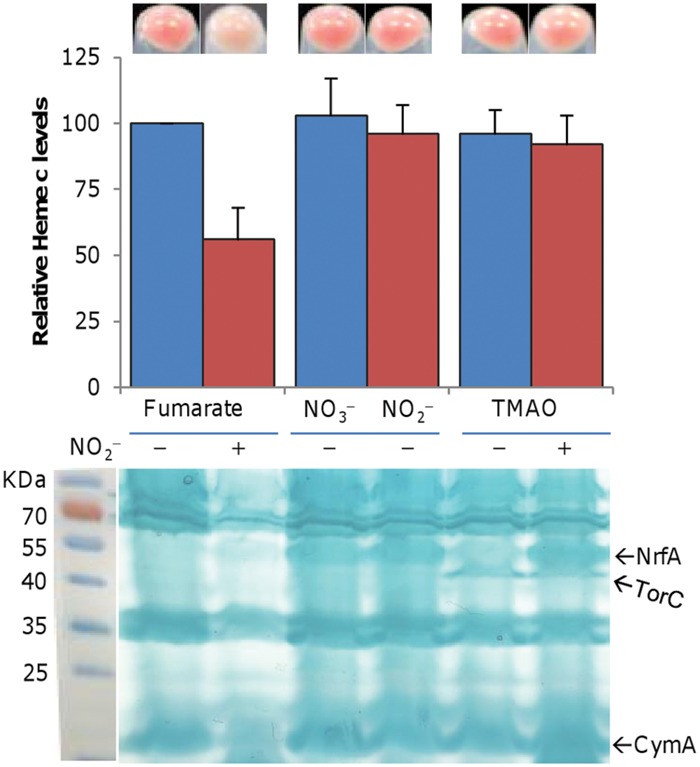
**Nitrite reduces levels of *c*-type cytochromes in cells grown on fumarate.** Mid-log phase cultures (~0.2 of OD_600_) grown on either fumarate or TMAO were pelletted and photographed (upper panel). Cell samples were lysed and the heme *c* levels were determined (middle panel). The average amount of heme *c* from the fumarate-grown samples without nitrite was set to 100%. Proteins (10 μg per lane) extracted from the indicated samples (with or without 2 mM nitrite) were resolved by SDS-PAGE and analyzed by heme staining (lower panel, a composite image in which a marker is placed next to the gel). Nitrite of 2 mM was used. Nitrate (2 mM) was included for comparison. The positions of NrfA, TorC, and CymA were marked. All experiments were performed at least three times with standard deviations presented as error bars or similar results were obtained.

The respiratory diversity of *S. oneidensis* is, at least in large part, due to its abundant *c*-type cytochromes, which confer to colonies or cell pellet an orange color (Meyer et al., 2004; Gao et al., 2010b). We noticed that the color of cell pellets grown on fumarate in the presence of nitrite was significantly lighter (**Figure 4**). Interestingly, this phenomenon was neither observed with either as sole EA nor with the TMAO/nitrite pair, implying that nitrite may reduce cytochrome *c* production in cells grown with fumarate but not TMAO. To test this possibility, we assayed heme *c* levels (**Figure 4**). The amount of total *c*-type cytochromes decreased significantly (to ~54% relative to the non-treated control) by including nitrite with fumarate but was not significantly altered with TMAO.

We then compared by SDS-PAGE the cytochrome *c* profiles of extracts of cells grown on fumarate or TMAO with or without nitrite and found that the overall amounts of *c*-type cytochromes were generally consistent with the pellet colors and the heme *c* levels (**Figure 4**). Three bands predicted to be NrfA, TorC, and CymA based on previous studies (Dos Santos et al., 1998; Myers and Myers, 2000; Dong et al., 2012) were excised, digested, and confirmed using mass spectrometry (MS). While TorC was visible as long as TMAO was present, NrfA showed a significantly different pattern. NrfA was evident in extracts of cells grown on nitrate, nitrite, or TMAO with nitrite but significantly weak in cells grown on TMAO only (Dong et al., 2012; Zhang et al., 2013). More importantly, this band was barely visible from extracts of cells grown on fumarate even in the presence of nitrite, suggesting that NrfA production is somehow blocked when both fumarate and nitrite are present.

### Reduced Production of *c*-Type Cytochromes in the Presence of Nitrite Is Partially Due to Reduced Crp-cAMP Activity

**FIGURE 5:**
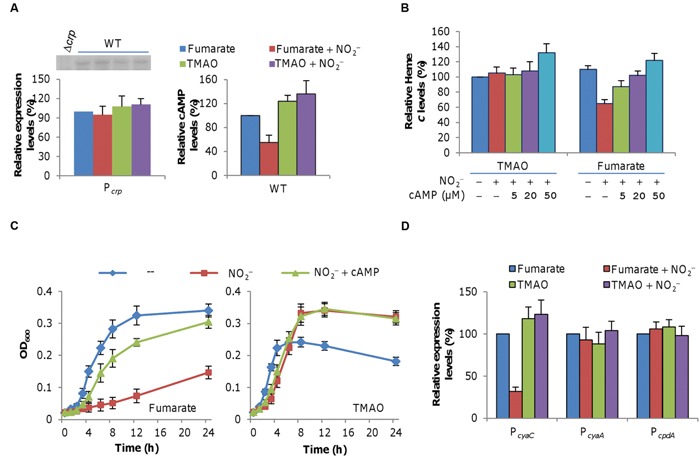
**Effect of exogenous cAMP on levels of *c*-type cytochromes and growth. (A)** Effect of nitrite on the expression of the *crp* gene (left panel) and cAMP levels (right panel) in cells grown on fumarate, fumarate and nitrite, TMAO, as well as TMAO and nitrite. Expression was assayed by using an integrative *lacZ* reporter as described in Section “Materials and Methods.” Results of western blotting of Crp were embedded with Δ*crp* as negative control (left panel). The average levels of expression (left panel) and cAMP (right panel) from the fumarate samples without nitrite were set to 100%. Cells were prepared as described in **Figure 4**. **(B)** Mid-log phase wild-type cultures under indicated conditions were collected and processed for determination of levels of *c*-type cytochromes as in **Figure 4**. **(C)** Growth of the wild-type under indicated conditions. cAMP concentration: 20 μM. Nitrite-free (–) cultures were included for comparison. **(D)** Expression of the genes involved in cAMP production (*cyaC* and *cyaA*) and degradation (*cpdA*) in cells grown on fumarate, fumarate and nitrite, TMAO, as well as TMAO and nitrite. All experiments were performed in triplicate and error bars indicate the standard error.

Previously, we had found that cells lacking global regulator Crp produced *c*-type cytochromes at a level similar to that for cells grown fumarate plus nitrite (Gao et al., 2010b), implying a link between nitrite treatment and Crp regulation. Therefore, we monitored expression of the *crp* gene with a *lacZ*-reporter. The *crp* promoter activity did not alter significantly in cells grown on fumarate with nitrite compared to those grown on just fumarate or for cells with TMAO in the absence or presence of nitrite (**Figure 5A**, left panel). Western blotting analysis of Crp revealed a similar result with a *crp* null mutant as control (embedded in **Figure 5A**). Given that the activity of Crp depends on formation of a Crp-cAMP complex (Charania et al., 2009; Zhou et al., 2013), we then assayed cAMP levels in these cells (**Figure 5A**, right panel). Compared to cells grown on fumarate in the absence of nitrite (which was set to 100%), those grown on TMAO with or without nitrite had significantly greater cAMP levels. In contrast, there was a pronounced decrease in cAMP in cells grown on fumarate with nitrite. These results suggest that the inhibitory effect of nitrite on growth in the presence of fumarate is, at least in part, due to a compromised Crp activity primarily by reducing intracellular cAMP levels.

To further confirm the involvement of the Crp-cAMP complex in the process, we examined effect of exogenous cAMP on cytochrome *c* levels and growth on fumarate or TMAO with nitrite. As shown in **Figure 5B**, the amounts of total *c*-type cytochromes in cells grown on TMAO and nitrite in the presence of cAMP up to 20 μM were similar but increased significantly (to ~138% relative to the nitrite-free control) when cAMP was supplemented to 50 μM. In contrast, the cAMP addition proportionally enhanced *c*-type cytochrome production in cells grown on fumarate plus nitrite, with restoration to the wild-type level being achieved by 20 μM. We then assessed whether exogenous cAMP also helps growth in the presence of nitrite (**Figure 5C**). Indeed, cAMP at 20 μM greatly improved growth on fumarate, which, however, was still significantly slow compared to the nitrite-free control. In the case of TMAO, effect of cAMP addition appeared negligible. While these data support the role of the Crp-cAMP complex in nitrite inhibition of growth on fumarate, it is clear that other factors are present.

In bacteria, cAMP is synthesized and degraded by ACs and phosphodiesterase, respectively (Botsford and Harman, 1992; Imamura et al., 1996). In *S. oneidensis*, there are three functional ACs, CyaA, CyaB, and CyaC, of which CyaC is the major AC for cAMP production and CyaA contributes slightly whereas CpdA is the only cAMP phosphodiesterase (Charania et al., 2009; Yin et al., 2016). To reveal direct factors for lowered cAMP levels in the presence of nitrite, we monitored expression of *cya* (*cyaA* and *cyaC*) and *cpdA* genes in the wild-type cultures grown on fumarate or TMAO w/o nitrite. As shown in **Figure 5D**, expression of *cyaC*, the major AC gene, but neither *cyaA* nor *cpdA*, was substantially repressed in the cells grown on both fumarate and nitrite, suggesting that the reduction in the cAMP amount is likely due to decreased production.

### Differential Expression of *nap* and *nrf* Operons in the Presence of Nitrite

**FIGURE 6:**
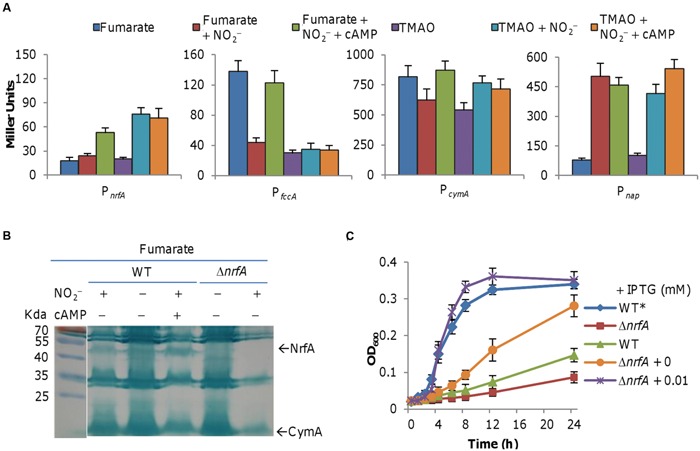
**Impacts of cAMP on expression of relevant genes. (A)** Effect of nitrite (2 mM) and cAMP (20 μM) on expression of relevant genes. **(B)** SDS-PAGE analysis of indicated strains grown on fumarate revealed that cAMP facilitates NrfA induction by nitrite. In both **(A,B)**, cells were prepared as described in **Figure 4**. This is a composite image in which a marker is placed next to the gel. **(C)** Growth of an *S. oneidensis* Δ*nrfA* mutant producing NrfA at varying levels on fumarate and nitrite. Growth of the wild-type on fumarate without nitrite (WT*) was included for comparison.

As shown above (**Figure 4**), nitrite is able to induce expression of *nrfA* in cells grown on TMAO but not on fumarate. To determine how reduced activity of the cAMP-Crp complex affects expression of relevant genes, we assayed their promoter activities by using an integrative *lacZ* reporter system (Fu et al., 2014). In the either absence or presence of nitrite, *nrfA* expression was extremely low in cells grown on fumarate but was induced more than threefold by nitrite in cells grown on TMAO (**Figure 6A**). In the case of *cymA* and *fccA* genes, both were downregulated by nitrite (**Figure 6A**). While the *fccA* gene had an approximately 70% decrease in expression, the expression reduction of the *cymA* gene in the presence of nitrite was less extensive (**Figure 6A**). We could not assess directly influence of the Crp loss on expression of these genes because the Δ*crp* strain could not grow on fumarate. As an alternative, we examined effect of cAMP addition on expression of these genes in cells grown on fumarate or TMAO w/o nitrite. Exogenous cAMP enhanced expression of the *nrfA*, *fccA*, and *cymA* genes in fumarate-grown cells when nitrite was present, which is in agreement with growth restoration by cAMP as shown in **Figures 5** and **6A**. Furthermore, these observations were supported in general by heme-staining data presented in **Figure 6B**, which included a verified *nrfA* null mutant (Δ*nrfA*) as negative control (Gao et al., 2009).

Given that forced expression of the *fccA* gene could not correct the growth defect in media containing fumarate and nitrite (**Figure 3D**), we reasoned that reduced production of NrfA underlies the growth defect. To test this, we first examined growth of the Δ*nrfA* strain on both fumarate and nitrite (Gao et al., 2009). Expectedly, it had further worsened growth than the wild-type (**Figure 6C**). We then introduced the *nrfA* gene placed under the control of P*_tac_* promoter into the wild-type and assayed growth. The effect was obvious. Without IPTG, growth was much improved because of expression from the leaky promoter (**Figure 6C**). In the presence of IPTG of 0.01 mM, with fumarate and nitrite cells carrying the construct displayed a growth pattern similar to that observed from cells grown on TMAO and nitrite (**Figure 6C**). These data thus confirm that the growth defect was due to low production of NrfA.

### Effects of Nitrite on cAMP Production Is Independent of NarP-NarQ

**FIGURE 7:**
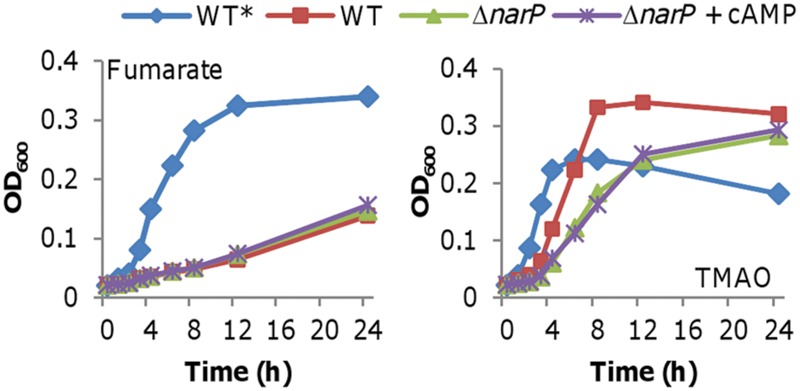
**Impacts of cAMP are independent of NarP-NarQ.** Growth of an *S. oneidensis narP* mutant on fumarate or TMAO with nitrite. This *narP* mutant was indistinguishable from the wild-type when grown fumarate or TMAO without nitrite (data not shown for clarity). Growth of the wild-type without nitrite (WT*) was included for comparison. cAMP concentration: 20 μM. All experiments were performed at least three times and error bars representing the standard error (<5%) were omitted for clarity.

In *S. oneidensis*, the NarP-NarQ two-component system (TCS) senses both nitrate and nitrite, and then regulates transcription of both the *nap* and *nrf* operons accordingly (Dong et al., 2012). We then tested whether the nitrite inhibition of cAMP production in cells grown on fumarate depends on NarP-NarQ. A *narP* null mutant (Δ*narP*), which is impaired in nitrite reduction (Dong et al., 2012), was indistinguishable from the wild-type when grown on fumarate and nitrite (**Figure 7**). In contrast, with TMAO and nitrite, this strain grew substantially slower than the wild-type, a phenotype in agreement with reduced NrfA production. Importantly, addition of exogenous cAMP had no significant effect when the TCS was absent (**Figure 7**). We then examined expression of the *nap* and *nrf* operons in a *narP* null mutant (Dong et al., 2012). In the Δ*narP* strain grown on either fumarate or TMAO, neither the *nap* nor the *nrf* operon was induced by nitrite with or without cAMP (data not shown). These results manifest that the TCS is the immediate regulator for the *nap* and *nrf* operons in response to nitrite while the cAMP-Crp complex functions at higher levels in the regulatory hierarchy.

## Discussion

Nitrite can enter the cytoplasm in the form of undissociated nitrous acid and through specific transporters (Samouilov et al., 2007; Lü et al., 2013). Despite this, it is likely that the majority of its targets reside in the periplasm, where it is at the highest concentrations. This may be particularly important for *S. oneidensis* as more than 40 *c*-type cytochromes are presented, either membrane-bound or soluble in the periplasm (Myers and Myers, 2000; Gao et al., 2010a; Maia and Moura, 2014). Nitrite inhibits the cytochrome *cbb*_3_ oxidase, the predominant system for oxygen respiration (Fu et al., 2013; Zhou et al., 2013). In addition, it has been reported that nitrite impedes respiration of soluble iron species in *S. oneidensis* (DiChristina, 1992). Furthermore, nitrite is more inhibitory anaerobically than aerobically and its targets under anoxic conditions remain undefined.

In this study, we aimed to unravel the mechanism of nitrite inhibition during anaerobiosis in *S. oneidensis*. By performing a series of experiments, we generated three important contributions to the current understanding of nitrite-associated physiology. First, we show that NO plays a negligible role in the phenotypes caused by nitrite. This is surprising because the antimicrobial action of nitrite has long been attributed to the formation of NO (Reddy et al., 1983; Hyduke et al., 2007; Mason et al., 2009; Richardson et al., 2011). Second, we present data to suggest that terminal reductases may not be direct targets of nitrite, contrasting the *cbb*_3_ oxidase for oxygen respiration (Fu et al., 2013). Third, we demonstrate that nitrite inhibition of growth on fumarate, presumably most of CymA-dependent EAs, is a result of reduced cAMP levels, leading to repression of both fumarate reductase and nitrite reductase.

**FIGURE 8:**
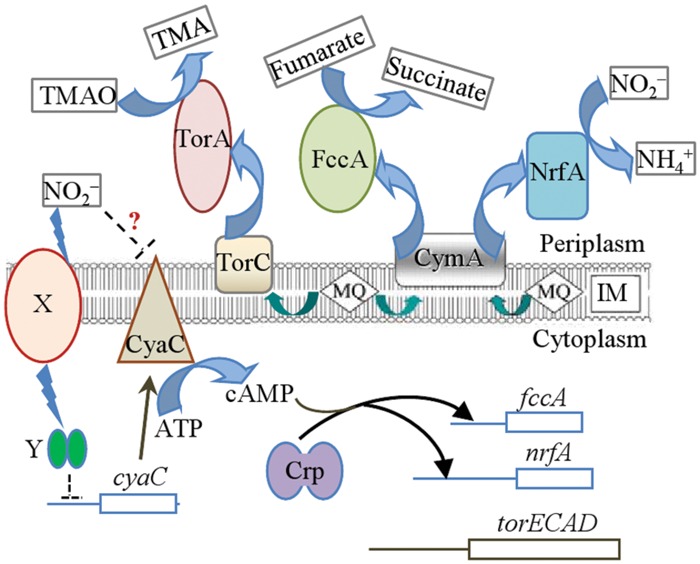
**Model for nitrite inhibition of CymA-dependent respiration in *S. oneidensis*.** Respiration of nitrite (NO_2_^-^) and fumarate but not TMAO is dependent on CymA. Nitrite triggers reduction in cAMP levels by diminishing *cyaC* expression through an unknown signal transduction cascade (X, signal recipient; Y, regulator) and probably also by inhibiting CyaC directly, which in turn represses *nrfA* and *fccA* expression. As a result, both enzymes at decreased levels impaired growth when both nitrite and fumarate are present. TMAO can overcome nitrite inhibition because its reductase is not under the control of cAMP-Crp.

Unlike the *E. coli* Crp which plays a central role in carbon catabolite repression (Görke and Stülke, 2008), the *S. oneidensis* counterpart is the predominant regulator controlling respiration (Saffarini et al., 2003; Gao et al., 2010b). Not surprisingly, a large number of genes involved in respiration including *nrfA*, *cymA*, and *fccA* are likely to be under direct control of Crp as Crp-binding motifs are predicted to be located in their promoter regions (Saffarini et al., 2003; Schwalb et al., 2003; Gao et al., 2010b; Novichkov et al., 2013). The present results show that nitrite compromises Crp activity by inhibiting production of the major AC CyaC and thereby reducing cAMP levels, resulting in the reduced expression of many *c*-type cytochrome genes (**Figure 8**). This notion is reasonable because lose of either of two cyclases that are responsible for cAMP production results in a substantial reduction in the activity of Crp (Charania et al., 2009). Moreover, overproduction of Crp could not fully complement a *crp* mutant (Fu et al., 2013) and cAMP in excess also affects Crp’s activity in *S. oneidensis* (Fu et al., 2013; Yin et al., 2016). Therefore, a strict stoichiometric ratio of Crp to cAMP molecules appears important for activity of Crp.

With low levels of cAMP, production of both fumarate and nitrite reductases lessens substantially in the presence of both fumarate and nitrite (**Figure 8**). Although the shortage of the former slows fumarate respiration and limits biomass yield, it is the reduced amount of the latter that is the determining factor for the growth defect, revealed by that the forced production of NrfA rather than FccA is able to correct the defect. In contrast, nitrite can be simultaneously utilized by cells respiring on TMAO, supporting significantly enhanced biomass. Unlike the reduction pathways for fumarate and soluble iron species as well as many others, the TMAO pathway does not use CymA as a quinol dehydrogenase (Marritt et al., 2012). Instead TMAO reduction depends on TorC, which along with the reductase, TorA, is not subject to Crp regulation (Gon et al., 2001; Gao et al., 2010b). Apparently, with TMAO supporting growth, intracellular cAMP levels can be maintained sufficiently high for NrfA production, allowing rapid removal of toxic nitrite.

The cAMP-Crp complex functions as an activator for both the *nap* and *nrf* operons, and so does NarP-NarQ TCS. The data presented here is in agreement with the previous proposal that the TCS is the immediate regulator for the *nap* and *nrf* operons while the cAMP-Crp complex functions at higher levels in the regulatory hierarchy (Dong et al., 2012). Intriguingly, although NarP-NarQ senses and responds to nitrite, it is not required for the role of nitrite in cAMP production. Exactly how nitrite triggers repression of cAMP production is not known. Additionally, unlike the *nrf* operon, the *nap* operon in cells grown on fumarate and nitrite is induced. Nitrite, like nitrate, is a strong inducer of the *napDAGHB* operon via the NarP-NarQ system provided that cAMP-Crp is present. The induction of the *nap* operon with low cAMP levels therefore suggests that its transcription does not depend on cAMP-Crp as strictly as that of the *nrf* operon. Coincidently, the predicted Crp-binding site for the *nap* operon is much less conserved than that for the *nrf* operon (Gao et al., 2010b; Novichkov et al., 2013). However, other possibilities may exist, and efforts to investigate these puzzles are underway.

How nitrite triggers reduction of cAMP biosynthesis remains unknown. As a matter of fact, despite intensive studies for decades we still know little about bacterial ability to maintain cAMP homeostasis in response to environmental and metabolic signals (Green et al., 2014). Crp has been a global protein under extensive investigation in the field of global transcription machinery engineering (gTME; Geng and Jiang, 2015). Numerous *E. coli crp* mutants showing improved performance under various stressful conditions, but the predominant mechanism for the elevated resistance is attributed to altered transcriptional profile, and stresses *per se* do not serve as a signal to mediate activity of cAMP-Crp (Geng and Jiang, 2015). Clearly, nitrite is different. We have previously suggested that regulation by cAMP-Crp is particularly critical in adaptation of *S. oneidensis* to redox-stratified environments. Although the major AC CyaC resides in the inner-membrane and is accessible to nitrite (Charania et al., 2009), nitrite may not directly inhibit the enzyme because the lowered cAMP concentration caused by nitrite seems to be more likely a result of reduced CyaC production (**Figure 8**). Nevertheless, this possibility will be tested in future. Crp is unlikely to be able to assume the signal-sensing role as it lacks redox-sensing domains (Fu et al., 2013; Zhou et al., 2013). Therefore, there must be some factors that sense redox signals (different EAs) and subsequently mediate cAMP levels (**Figure 8**). Identification of such factors is undoubtedly a significant challenge. Nevertheless, our data indicate that nitrite could serve as a good signal for unraveling the signal transduction cascade, which is under way.

Intriguingly, both CymA and FccA display a dose-dependent impact on cells grown on fumarate. FccA is a unique fumarate reductase located in the periplasm, and CymA is responsible for electron transfer to FccA from the quinol pool (Leys et al., 1999). Although, further investigation is needed, a recent finding offers a feasible explanation (McMillan et al., 2013). It is known that the quinol dehydrogenase activity of CymA is triggered by formation of a CymA-FccA complex. Respiratory electron flux relies to some extent on the stability of the complexes. Without FccA, CymA could only reduce the quinol species, menaquinol-7. Hence, the formation of the complex regulates the activity of CymA by changing the direction of electron flow. Accordingly, we speculate that optimum respiration depends on a certain stoichiometric ratio of CymA to FccA molecules and overproducing either would undermine the interaction, leading to the impaired respiration of fumarate.

## Author Contributions

Jie Yuan and HG conceived the idea and designed the project. MJ, HF, and Jianhua Yin carried out the experiments. MJ and HG analyzed data. MJ and HG wrote the paper.

## Conflict of Interest Statement

The authors declare that the research was conducted in the absence of any commercial or financial relationships that could be construed as a potential conflict of interest.

## References

[B1] Botsford J. L. Harman J. G. (1992). Cyclic AMP in prokaryotes. *Microbiol. Rev.* 56 100–122. 1315922 10.1128/mr.56.1.100-122.1992 PMC372856

[B2] Charania M. A. Brockman K. L. Zhang Y. Banerjee A. Pinchuk G. E. Fredrickson J. K. (2009). Involvement of a membrane-bound class III adenylate cyclase in regulation of anaerobic respiration in *Shewanella oneidensis* MR-1. *J. Bacteriol.* 191 4298–4306. 10.1128/JB.01829-08 19395492 PMC2698484

[B3] Chen H. Luo Q. Yin J. Gao T. Gao H. (2015). Evidence for the requirement of CydX in function but not assembly of the cytochrome bd oxidase in *Shewanella oneidensis*. *Biochim. Biophys. Acta* 1850 318–328. 10.1016/j.bbagen.2014.10.005 25316290

[B4] Cordova C. D. Schicklberger M. F. R. Yu Y. Spormann A. M. (2011). Partial functional replacement of CymA by SirCD in *Shewanella oneidensis* MR-1. *J. Bacteriol.* 193 2312–2321. 10.1128/JB.01355-10 21378180 PMC3133100

[B5] Cruz-Garcia C. Murray A. E. Klappenbach J. A. Stewart V. Tiedje J. M. (2007). Respiratory nitrate ammonification by *Shewanella oneidensis* MR-1. *J. Bacteriol.* 189 656–662. 10.1128/JB.01194-06 17098906 PMC1797406

[B6] DiChristina T. J. (1992). Effects of nitrate and nitrite on dissimilatory iron reduction by *Shewanella putrefaciens* 200. *J. Bacteriol.* 174 1891–1896. 1548235 10.1128/jb.174.6.1891-1896.1992 PMC205793

[B7] Dong Y. Wang J. Fu H. Zhou G. Shi M. Gao H. (2012). A Crp-Dependent two-component system regulates nitrate and nitrite respiration in *Shewanella oneidensis*. *PLoS ONE* 7:e51643 10.1371/journal.pone.0051643 PMC3519889 23240049

[B8] Dos Santos J.-P. Iobbi-Nivol C. Couillault C. Giordano G. Méjean V. (1998). Molecular analysis of the trimethylamine N-oxide (TMAO) reductase respiratory system from a *Shewanella* species. *J. Mol. Biol.* 284 421–433. 10.1006/jmbi.1998.2155 9813127

[B9] Fredrickson J. K. Romine M. F. Beliaev A. S. Auchtung J. M. Driscoll M. E. Gardner T. S. (2008). Towards environmental systems biology of *Shewanella*. *Nat. Rev. Micro.* 6 592–603. 10.1038/nrmicro1947 18604222

[B10] Fu H. Chen H. Wang J. Zhou G. Zhang H. Zhang L. (2013). Crp-dependent cytochrome bd oxidase confers nitrite resistance to *Shewanella oneidensis*. *Environ. Microbiol.* 15 2198–2212. 10.1111/1462-2920.12091 23414111

[B11] Fu H. Jin M. Ju L. Mao Y. Gao H. (2014). Evidence for function overlapping of CymA and the cytochrome bc1 complex in the *Shewanella oneidensis* nitrate and nitrite respiration. *Environ. Microbiol.* 16 3181–3195. 10.1111/1462-2920.12457 24650148

[B12] Fu H. Jin M. Wan F. Gao H. (2015). *Shewanella oneidensis* cytochrome c maturation component CcmI is essential for heme attachment at the non-canonical motif of nitrite reductase NrfA. *Mol. Microbiol.* 95 410–425. 10.1111/mmi.12865 25402661

[B13] Gao H. Barua S. Liang Y. Wu L. Dong Y. Reed S. (2010a). Impacts of *Shewanella oneidensis* c-type cytochromes on aerobic and anaerobic respiration. *Microb. Biotechnol.* 3 455–466. 10.1111/j.1751-7915.2010.00181.x 21255343 PMC3815811

[B14] Gao H. Wang X. Yang Z. K. Chen J. Liang Y. Chen H. (2010b). Physiological roles of ArcA, Crp, and EtrA and their interactive control on aerobic and anaerobic respiration in *Shewanella oneidensis*. *PLoS ONE* 5:e15295 10.1371/journal.pone.0015295 PMC3011009 21203399

[B15] Gao H. Yang Z. K. Barua S. Reed S. B. Romine M. F. Nealson K. H. (2009). Reduction of nitrate in *Shewanella oneidensis* depends on atypical NAP and NRF systems with NapB as a preferred electron transport protein from CymA to NapA. *ISME J.* 3 966–976. 10.1038/ismej.2009.40 19387485

[B16] Gao T. Ju L. Yin J. Gao H. (2015). Positive regulation of the *Shewanella oneidensis* OmpS38, a major porin facilitating anaerobic respiration, by Crp and Fur. *Sci. Rep.* 5 14263 10.1038/srep14263 PMC4585640 26381456

[B17] Geng H. Jiang R. (2015). cAMP receptor protein (CRP)-mediated resistance/tolerance in bacteria: mechanism and utilization in biotechnology. *Appl. Micriobiol. Biotechnol.* 99 4533–4543. 10.1007/s00253-015-6587-0 25913005

[B18] Goldstein S. Russo A. Samuni A. (2003). Reactions of PTIO and Carboxy-PTIO with ⋅NO ⋅NO2 and O2-⋅ *J. Biol. Chem.* 278 50949–50955. 10.1074/jbc.M308317200 12954619

[B19] Gon S. Giudici-Orticoni M.-T. Méjean V. Iobbi-Nivol C. (2001). Electron transfer and binding of the c-type cytochrome TorC to the trimethylamine n-oxide reductase in *Escherichia coli*. *J. Biol. Chem.* 276 11545–11551. 10.1074/jbc.M008875200 11056172

[B20] Gon S. Patte J.-C. Dos Santos J.-P. Méjean V. (2002). Reconstitution of the trimethylamine oxide reductase regulatory elements of *Shewanella oneidensis* in *Escherichia coli*. *J. Bacteriol.* 184 1262–1269. 10.1128/JB.184.5.1262-1269.2002 11844754 PMC134858

[B21] Görke B. Stülke J. (2008). Carbon catabolite repression in bacteria: many ways to make the most out of nutrients. *Nat. Rev. Micro.* 6 613–624. 10.1038/nrmicro1932 18628769

[B22] Gralnick J. A. Vali H. Lies D. P. Newman D. K. (2006). Extracellular respiration of dimethyl sulfoxide by *Shewanella oneidensis* strain MR-1. *Proc. Natl. Acad. Sci. U.S.A.* 103 4669–4674. 10.1073/pnas.0505959103 16537430 PMC1450229

[B23] Green J. Stapleton M. R. Smith L. J. Artymiuk P. J. Kahramanoglou C. Hunt D. M. (2014). Cyclic-AMP and bacterial cyclic-AMP receptor proteins revisited: adaptation for different ecological niches. *Curr. Opin. Microbiol.* 18 1–7. 10.1016/j.mib.2014.01.003 24509484 PMC4005916

[B24] Hyduke D. R. Jarboe L. R. Tran L. M. Chou K. J. Y. Liao J. C. (2007). Integrated network analysis identifies nitric oxide response networks and dihydroxyacid dehydratase as a crucial target in *Escherichia coli*. *Proc. Natl. Acad. Sci. U.S.A.* 104 8484–8489. 10.1073/pnas.0610888104 17494765 PMC1895976

[B25] Imamura R. Yamanaka K. Ogura T. Hiraga S. Fujita N. Ishihama A. (1996). Identification of the cpdA gene encoding cyclic 3’,5’-adenosine monophosphate phosphodiesterase in *Escherichia coli*. *J. Biol. Chem.* 271 25423–25429. 10.1074/jbc.271.41.25423 8810311

[B26] Jepson B. J. N. Marietou A. Mohan S. Cole J. A. Butler C. S. Richardson D. J. (2006). Evolution of the soluble nitrate reductase: defining the monomeric periplasmic nitrate reductase subgroup. *Biochem. Soc. Trans.* 34 122–126. 10.1042/BST0340122 16417499

[B27] Jin M. Jiang Y. Sun L. Yin J. Fu H. Wu G. (2013). Unique organizational and functional features of the cytochrome c maturation system in *Shewanella oneidensis*. *PLoS ONE* 8:e75610 10.1371/journal.pone.0075610 PMC3769277 24040415

[B28] Leys D. Tsapin A. S. Nealson K. H. Meyer T. E. Cusanovich M. A. Beeumen J. J. V. (1999). Structure and mechanism of the flavocytochrome c fumarate reductase of *Shewanella putrefaciens* MR-1. *Nat. Struct. Mol. Biol.* 6 1113–1117. 10.1038/70051 10581551

[B29] Lü W. Du J. Schwarzer Nikola J. Wacker T. Andrade Susana L. A. Einsle O. (2013). The formate/nitrite transporter family of anion channels. *Biol. Chem.* 394 715–727. 10.1515/hsz-2012-0339 23380538

[B30] Luo Q. Dong Y. Chen H. Gao H. (2013). Mislocalization of Rieske protein PetA predominantly accounts for the aerobic growth defect of tat mutants in *Shewanella oneidensis*. *PLoS ONE* 8:e62064 10.1371/journal.pone.0062064 PMC3623810 23593508

[B31] Maia L. B. Moura J. J. G. (2014). How biology handles nitrite. *Chem. Rev.* 114 5273–5357. 10.1021/cr400518y 24694090

[B32] Maier T. M. Myers J. M. Myers C. R. (2003). Identification of the gene encoding the sole physiological fumarate reductase in *Shewanella oneidensis* MR-1. *J. Basic Microbiol.* 43 312–327. 10.1002/jobm.200390034 12872312

[B33] Marritt S. J. Lowe T. G. Bye J. McMillan D. G. G. Shi L. Fredrickson J. (2012). A functional description of CymA, an electron-transfer hub supporting anaerobic respiratory flexibility in *Shewanella*. *Biochem. J.* 444 465–474. 10.1042/BJ20120197 22458729

[B34] Mason M. G. Shepherd M. Nicholls P. Dobbin P. S. Dodsworth K. S. Poole R. K. (2009). Cytochrome bd confers nitric oxide resistance to *Escherichia coli*. *Nat. Chem. Biol.* 5 94–96. 10.1038/nchembio.135 19109594

[B35] McMillan D. G. G. Marritt S. J. Firer-Sherwood M. A. Shi L. Richardson D. J. Evans S. D. (2013). Protein–protein interaction regulates the direction of catalysis and electron transfer in a redox enzyme complex. *J. Am. Chem. Soc.* 135 10550–10556. 10.1021/ja405072z 23799249 PMC3823026

[B36] Meyer T. E. Tsapin A. I. Vandenberghe I. De Smet L. Frishman D. Nealson K. H. (2004). Identification of 42 possible cytochrome c genes in the *Shewanella oneidensis* genome and characterization of six soluble cytochromes. *OMICS* 8 57–77. 10.1089/153623104773547499 15107237

[B37] Miranda K. M. Espey M. M. G. Wink D. A. A. (2001). A rapid, simple spectrophotometric method for simultaneous detection of nitrate and nitrite. *Nitric Oxide* 5 62–71. 10.1006/niox.2000.0319 11178938

[B38] Myers J. M. Myers C. R. (2000). Role of the tetraheme cytochrome CymA in anaerobic electron transport in cells of *Shewanella putrefaciens* MR-1 with normal levels of menaquinone. *J. Bacteriol.* 182 67–75. 10.1128/JB.182.1.67-75.2000 10613864 PMC94241

[B39] Novichkov P. Kazakov A. Ravcheev D. Leyn S. Kovaleva G. Sutormin R. (2013). RegPrecise 3.0 *–* a resource for genome-scale exploration of transcriptional regulation in bacteria. *BMC Genomics* 14:745 10.1186/1471-2164-14-745 PMC3840689 24175918

[B40] Plate L. Marletta M. A. (2012). Nitric oxide modulates bacterial biofilm formation through a multicomponent cyclic-di-GMP signaling network. *Mol. Cell* 46 449–460. 10.1016/j.molcel.2012.03.023 22542454 PMC3361614

[B41] Price M. S. Chao L. Y. Marletta M. A. (2007). *Shewanella oneidensis* MR-1 H-NOX regulation of a histidine kinase by nitric oxide. *Biochemistry* 46 13677–13683. 10.1021/bi7019035 17988156 PMC2531215

[B42] Reddy D. Lancaster J. Cornforth D. (1983). Nitrite inhibition of *Clostridium botulinum*: electron spin resonance detection of iron-nitric oxide complexes. *Science* 221 769–770. 10.1126/science.6308761 6308761

[B43] Richardson A. R. Payne E. C. Younger N. Karlinsey J. E. Thomas V. C. Becker L. A. (2011). Multiple targets of nitric oxide in the tricarboxylic acid cycle of *Salmonella enterica* serovar typhimurium. *Cell Host Microbe* 10 33–43. 10.1016/j.chom.2011.06.004 21767810 PMC3142370

[B44] Saffarini D. A. Schultz R. Beliaev A. (2003). Involvement of cyclic AMP (cAMP) and cAMP receptor protein in anaerobic respiration of *Shewanella oneidensis*. *J. Bacteriol.* 185 3668–3671. 10.1128/JB.185.12.3668-3671.2003 12775705 PMC156221

[B45] Samouilov A. Woldman Y. Y. Zweier J. L. Khramtsov V. V. (2007). Magnetic resonance study of the transmembrane nitrite diffusion. *Nitric Oxide* 16 362–370. 10.1016/j.niox.2006.12.006 17306575 PMC2709508

[B46] Schwalb C. Chapman S. K. Reid G. A. (2003). The tetraheme cytochrome CymA is required for anaerobic respiration with dimethyl sulfoxide and nitrite in *Shewanella oneidensis*. *Biochemistry* 42 9491–9497. 10.1021/bi034456f 12899636

[B47] Shi L. Rosso K. M. Clarke T. A. Richardson D. J. Zachara J. M. Fredrickson J. K. (2012). Molecular underpinnings of Fe(III) oxide reduction by *Shewanella oneidensis* MR-1. *Front. Microbiol.* 3:50 10.3389/fmicb.2012.00050 PMC3279761 22363328

[B48] Shi M. Gao T. Ju L. Yao Y. Gao H. (2014). Effects of FlrBC on flagellar biosynthesis of *Shewanella oneidensis*. *Mol. Microbiol.* 93 1269–1283. 10.1111/mmi.12731 25074236

[B49] Shi M. Wan F. Mao Y. Gao H. (2015). Unraveling the mechanism for the viability deficiency of *Shewanella oneidensis* oxyR null mutant. *J. Bacteriol.* 197 2179–2189. 10.1128/JB.00154-15 25897035 PMC4455265

[B50] Shirodkar S. Reed S. Romine M. Saffarini D. (2011). The octahaem SirA catalyses dissimilatory sulfite reduction in *Shewanella oneidensis* MR-1. *Environ. Microbiol.* 13 108–115. 10.1111/j.1462-2920.2010.02313.x 21199252

[B51] Simpson P. J. L. Richardson D. J. Codd R. (2010). The periplasmic nitrate reductase in *Shewanella*: the resolution, distribution and functional implications of two NAP isoforms, NapEDABC and NapDAGHB. *Microbiology* 156 302–312. 10.1099/mic.0.034421-0 19959582

[B52] Sun L. Dong Y. Shi M. Jin M. Zhou Q. Luo Z. Q. (2014). Two residues predominantly dictate functional difference in motility between *shewanella oneidensis* flagellins FlaA and FlaB. *J. Biol. Chem.* 289 14547–14559. 10.1074/jbc.M114.552000 24733391 PMC4031512

[B53] Sun L. Jin M. Ding W. Yuan J. Kelly J. Gao H. (2013). Post-translational modification of flagellin FlaB in *Shewanella oneidensis*. *J. Bacteriol.* 195 2550–2561. 10.1128/JB.00015-13 23543712 PMC3676071

[B54] Thomas P. E. Ryan D. Levin W. (1976). An improved staining procedure for detection of peroxidase activity of cytochrome P-450 on sodium dodecyl sulphate polyacrylamide gels. *Anal. Biochem.* 75 168–176. 10.1016/0003-2697(76)90067-1 822747

[B55] Yin J. Jin M. Zhang H. Ju L. Zhang L. Gao H. (2015). Regulation of nitrite resistance of the cytochrome cbb3 oxidase by cytochrome c ScyA in *Shewanella oneidensis*. *Microbiologyopen* 4 84–99. 25417822 10.1002/mbo3.224 PMC4335978

[B56] Yin J. Meng Q. Fu H. Gao H. (2016). Reduced expression of cytochrome oxidases largely explains cAMP inhibition of aerobic growth in *Shewanella oneidensis*. *Sci. Rep.* 6 24449 10.1038/srep24449 PMC4830989 27076065

[B57] You C. Okano H. Hui S. Zhang Z. Kim M. Gunderson C. W. (2013). Coordination of bacterial proteome with metabolism by cyclic AMP signalling. *Nature* 500 301–306. 10.1038/nature12446 23925119 PMC4038431

[B58] Zhang H. Fu H. Wang J. Sun L. Jiang Y. Zhang L. (2013). Impacts of nitrate and nitrite on physiology of *Shewanella oneidensis*. *PLoS ONE* 8:e62629 10.1371/journal.pone.0062629 PMC3633839 23626841

[B59] Zhou G. Yin J. Chen H. Hua Y. Sun L. Gao H. (2013). Combined effect of loss of the *caa*_3_ oxidase and Crp regulation drives *Shewanella* to thrive in redox-stratified environments. *ISME J.* 7 1752–1763. 10.1038/ismej.2013.62 23575370 PMC3749501

