# Supplementary material for: Molecular Underpinnings of Nitrite Effect on CymA-Dependent Respiration in Shewanella oneidensis
Source: Front Microbiol. 2016 Jul 21;7:1154. doi: 10.3389/fmicb.2016.01154 (PMC4954811; doi:10.3389/fmicb.2016.01154)
Supplement: Supplementary file 1 [file Presentation_1.PDF]

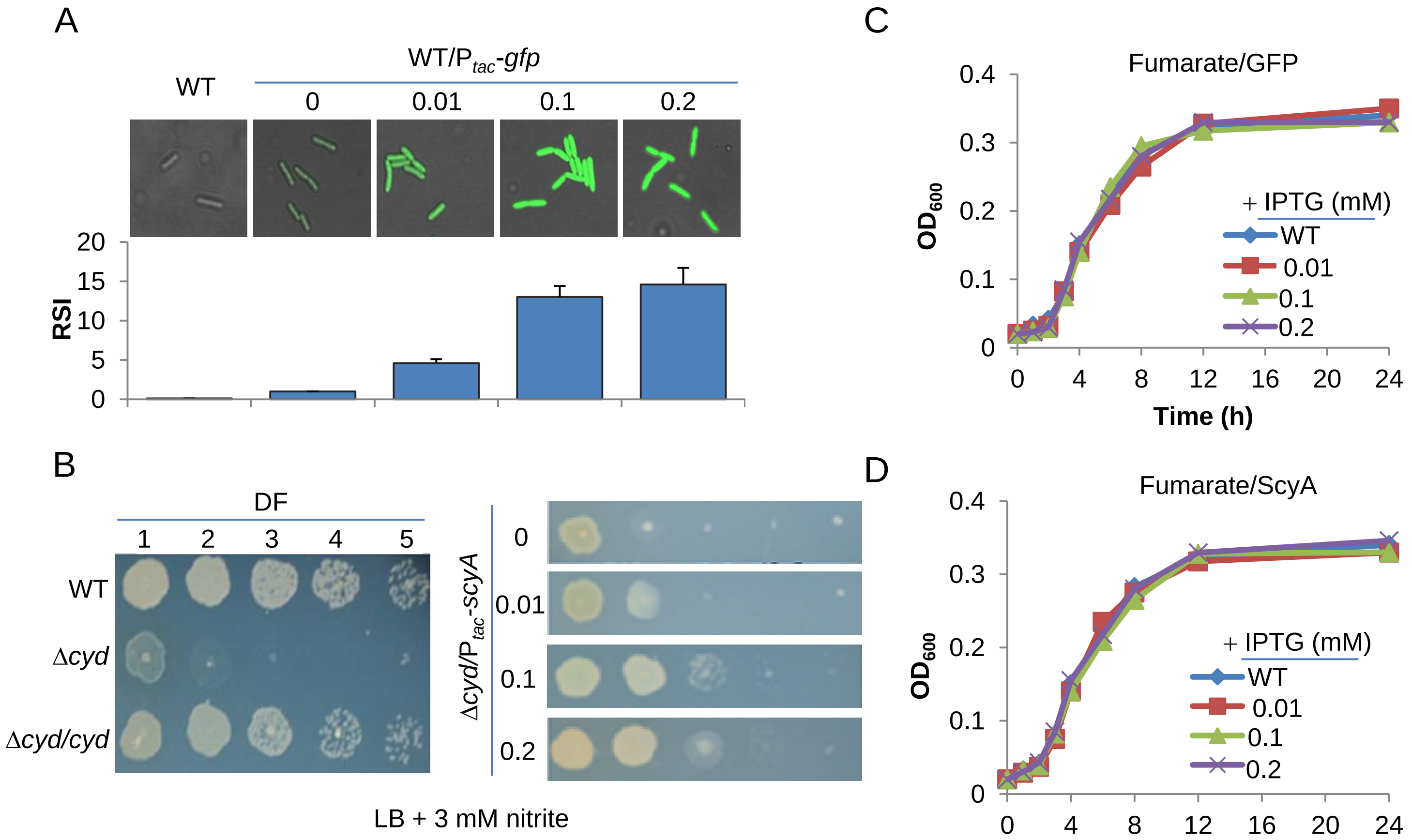

Figure S1. Expression of the *gfp* and *scyA* genes driven by P_{tac} within pHGE-P*tac*. Note that the promoter is slightly leaky. (**A**) Expression of *gfp* under control of IPTG. Levels of GFP were visualized and photographed with a confocal microscope. Signal intensities were quantified as described in Experimental procedures and presented as relative levels, normalizing to the average of sample without IPTG, which was set to 1. (**B**) Expression of *scyA* under control of IPTG. In *S. oneidensis*, cytochrome *cbb*$_3$ oxidase is highly sensitive to nitrite, and thus loss of alternative *bd* oxidase heavily impairs ability to grow in the presence of 3 mM nitrite under aerobic conditions. ScyA in excess elevates the nitrite resistance of the *cbb*$_3$ oxidase, improving growth of Δ*cyd* against nitrite, which is in excellent agreement with the results of a previous study. Expression of the *gfp* and *scyA* genes was driven by P_{tac}, which is under control of IPTG, within pHGE-Ptac. Numbers in each panel represent levels of IPTG (mM) added. For comparison, WT carried empty plasmid. (**C**) Effect of overproduced GFP on growth on fumarate. (**D**) Effect of overproduced ScyA on growth on fumarate. In panels (**C**) and (**D**), error bars representing standard deviations from at least three independent experiments, similar to those presented in Fig. 1 and 2, were omitted for clarity.
